# Prediction of asthma using a four-locus gene model including *IL13*, *IL4*, *FCER1B*, and *ADRB2* in children of Kazak nationality

**DOI:** 10.1186/s13052-023-01564-y

**Published:** 2023-12-04

**Authors:** Shasha Bai, Jun Lu, Li Hua, Quanhua Liu, Mengxue Chen, Yutong Gu, Jianhua Zhang, Dongjun Ma, Yixiao Bao

**Affiliations:** 1 Department of Pediatric Pulmonology, Xinhua Hospital Affiliated to Shanghai Jiao Tong University School of Medicine, Shanghai, China; 2 Children’s Respiratory Diagnosis and Treatment Center, Urumqi First People’s Hospital, Xinjiang, China

**Keywords:** Gene model, Genotype, Allele, Asthma, Kazak

## Abstract

**Background:**

To study whether the four locus gene model consisting of *ADRB2* rs1042713, *IL4* rs2243250, *FCER1B* rs569108 and *L13* rs20541 can predict asthma of the Kazak children in Xinjiang, China.

**Methods:**

Four single nucleotide polymorphisms about the 4 genes were genotyped in asthma group and control group of Han children and Kazak children respectively. The frequencies of different genotypes and alleles were compared between the asthma group and the control group in the two nationalities. Different risk genotypes for asthma were evaluated in the two nationalities.

**Results:**

The differences about frequencies of genotypes in *ADRB2* rs1042713 and *IL4* rs2243250 and *IL13* rs20541 between asthma group and control group were statistically significant in Han children, as were the frequencies of alleles in the 3 single nucleotide polymorphisms, but there were no statistical differences in *FCER1B* rs569108(*P* > 0.05). For the Kazak children, no differences were existed among all the genotypes and alleles in asthma group and control group. For the Han children, more children were asthma high risk genotype in the asthma group than those in the control group and no difference was found in the Kazak children.

**Conclusions:**

The four locus gene model consisting of *ADRB2* rs1042713, *IL4* rs2243250, *FCER1B* rs569108 and *L13* rs20541 can predict asthma of Han children but not for the Kazak children in Xinjiang, which illustrating that the difference of asthma prevalence between different races is closely related to the genetic background.

## Introduction

Asthma is a polygenic disease with complex phenotypes. There are significant racial differences in the prevalence of childhood asthma. In America, Puerto Ricans have the highest incidence of asthma, followed by African Americans, and Mexican children have the lowest incidence of asthma [1]. Compared with the native Americans, the genetic susceptibility of African Americans to asthma is associated with increased disease severity, increased blood IgE levels, and decreased lung function. Genome-wide association studies have found the race-specific genetic variants [2, 3]. In addition, researchers used cluster analysis to find that there were genetic variants related to the clinical phenotype of asthma in African American children [4]. The third survey of asthma prevalence among children aged 0–14 years in China in 2010 showed that the prevalence of asthma in children of different ethnic groups is significantly different. The prevalence of Han children is the highest at 3%, while the prevalence of children of Tibetan is the lowest, which is only 0.52% [5]. The prevalence of asthma among Uyghur children is much lower than Han children in the same region. Zhang et al. found that the difference in the prevalence of asthma among children of this ethnic group may be related to the gene *IL-4* -590C > T [6]. Our previous case–control research found that *IL-13* C1923T T/T is associated with Mauritian Indian children’s asthma, but not with Han children [7], which was consistent with the results of Leung TF et al. [8].

However, the research on the correlation between the prevalence of asthma in children of different races and the susceptibility gene is mostly focused on the poly- morphism of a single locus, and the correlation between multiple SNP loci and even the gene prediction models are rarely conducted. So finding out the SNP loci of the associated genes has important scientific significance for understanding the differences in asthma prevalence among different ethnic groups. As we all know, China is a multi-ethnic country, and Xinjiang is a place where ethnic minorities live together. In addition to the large number of Uyghurs, there are 47 ethnic groups such as Kazakh, Hui, and Mongolian. The genetic resources are very rich. The Kazakh ethnic group in Xinjiang still retains a relatively complete isolated genetic system and is an ideal population for conducting genetic research on complex diseases.

The establishment and verification of the gene model for predicting children's asthma susceptibility previously carried out by our group are from Han children [9, 10]. It is still unclear whether the model is also applicable to other ethnic groups in China. In this study, the gene prediction model was tested in the Han and Kazakh children in Xinjiang, and the four SNP loci included in the gene model were compared: ADRB2 rs1042713, IL4 rs2243250, FCER1B rs569108 and IL13 rs20541 with differences among different ethnic groups. In addition, further analysis about the differences in the distribution of different asthma risk genotypes basing on the model in the two ethnic groups will be carried out, which may provide a scientific basis for finding the causes of asthma-related susceptibility genes in Kazakh children and differences in the prevalence of asthma among different ethnic groups.

## Methods

### Study population

Our study enrolled 181 children including the Han and Kazak younger than 14 years old, who were diagnosed asthma in the First People's Hospital of Urumqi, Xinjiang, China, as well as 185 children in the control group.

The study inclusion criteria consisted of the following: The diagnostic criteria of asthma refers to the 2016 edition of the diagnostic guidelines for children with bronchial asthma formulated by the Respiratory Group of the Pediatric Branch of the Chinese Medical Association; Children in the control group are required to have no personal history of eczema, as well as have no allergic rhinitis and wheezing, not any food allergy, and no family history of allergic diseases. To exclude the influence of environmental factors, all children are required to live in Xinjiang for at least 3 generations. The exclusion criteria were: congenital pulmonary disease, congenital heart disease, congenital vascular malformation, congenital immune defects, foreign body aspiration, and tuberculosis of the bronchial lymph nodes. We obtained written informed consent from their guardians. The study was approved by the Ethics Committee of the hospital and was conducted according to the principles of the Declaration of Helsinki.

### Genotyping and grouping

The genomic DNA of the four genes were extracted from buccal mucosa using a DNA extracting kit (Emer Ther). The genotypes of the 4 gene loci were obtained by the methods of Multiplex polymerase chain reaction (PCR). The PCR cycling conditions were as follows: 95 °C for 2 min; 45 cycles at 95 °C for 30 s, 56 °C for 30 s, and 72 °C for 60 s; and finally, 72 °C for 5 min. The conditions for the SAP enzyme digestion reaction were 37 °C for 40 min and then termination at 85 °C for 5 min. The conditions used for iPLEX were 95 °C for 30 s; 5 inner cycles at 52 °C for 5 s and at 85 °C for 5 s; and 40 outer cycles at 94 °C for 5 s, 52 °C for 5 s, and 85 °C for 5 s. Then we used matrix-assisted laser desorption ionization time-of-flight mass spectrometry to discriminate between the 4 SNPs [10].

In our study, the different risk genotypes for asthma were discriminated according to the number of risk allele homozygotes. The control group comprised patients with no risk homozygotes (ie IL13 rs20541 AA or GA, IL4 rs2243250 CC or TC, ADRB2 rs1042713 GG or AG, and FCER1B rs569108 AA or AG). Those with different genotype combinations who had a significantly higher risk of asthma (if *P* < 0.05 and OR > 1) than the reference group were defined as being at higher genetic risk for asthma. The others were defined as being at lower genetic risk for asthma. The grouping results of the different genotype combinations were shown in Table 1.

**Table 1:** High- and low-risk genotype of asthma according to the 4 genotypes

Groups	Genotype			
	IL13 rs20541	IL4 rs2243250	ADRB2 rs1042713	FCER1B rs569108
Low-risk genotype	AA or GA	CC or TC	GG or AG	AA or AG
	AA or GA	CC or TC	GG or AG	GG
	AA or GA	CC or TC	AA	AA or AG
	AA or GA	TT	GG or AG	AA or AG
	GG	CC or TC	GG or AG	AA or AG
	AA or GA	CC or TC	AA	GG
	GG	CC or TC	GG or AG	GG
High-risk genotype	AA or GA	TT	GG or AG	GG
	AA or GA	TT	AA	AA or AG
	GG	CC or TC	AA	AA or AG
	GG	TT	GG or AG	AA or AG
	AA or GA	TT	AA	GG
	GG	CC or TC	AA	GG
	GG	TT	GG or AG	GG
	GG	TT	AA	AA or AG
	GG	TT	AA	GG

### Statistical analysis

Calculating the allele frequency and genotype frequency.

N represents the number of samples, A and B represent two risk alleles, AA, AB and BB represent three genotypes, and the number of each genotype is represented by N_AA_, N_AB_ and N_BB_ respectively. Then

$$\mathrm{A}= ({\mathrm{N}}_{\mathrm{AA}}+ {\mathrm{N}}_{\mathrm{AB}} /2) / ({\mathrm{N}}_{\mathrm{AA}} + {\mathrm{N}}_{\mathrm{AB}}+ {\mathrm{N}}_{\mathrm{BB}})$$

$$\mathrm{B}= ({\mathrm{N}}_{\mathrm{BB}}+ {\mathrm{N}}_{\mathrm{AB}} /2) / ({\mathrm{N}}_{\mathrm{AA}} + {\mathrm{N}}_{\mathrm{AB}}+ {\mathrm{N}}_{\mathrm{BB}})$$

$$\mathrm{AA}= \left({\mathrm{N}}_{\mathrm{AA}}\right) / ( {\mathrm{N}}_{\mathrm{AA}} + {\mathrm{N}}_{\mathrm{AB}} + {\mathrm{N}}_{\mathrm{BB}})$$

$$\mathrm{AB}= \left({\mathrm{N}}_{\mathrm{AB}}\right) / ( {\mathrm{N}}_{\mathrm{AA}} + {\mathrm{N}}_{\mathrm{AB}} + {\mathrm{N}}_{\mathrm{BB}})$$

$$\mathrm{BB}= \left({\mathrm{N}}_{\mathrm{BB}}\right) / ( {\mathrm{N}}_{\mathrm{AA}} + {\mathrm{N}}_{\mathrm{AB}} + {\mathrm{N}}_{\mathrm{BB}})$$

All statistical analyses were conducted using the SPSS package version 20.0 (IBM Corporation, USA). The measurement data was expressed as mean ± standard deviation. The mean of the two samples was compared by Student's t test or Rank sum test. Genotype frequency and allele frequency were calculated by gene counting method. Pearson χ^2^ test or Fisher exact probability method were used to compare the difference of allele frequency, genotype frequency and the distribution of high and low risk types of asthma between the asthma group and control group. Two-tailed P values of 0.05 or less were considered to be statistically significant.

## Results

### The distribution of basic information of the Han and Kazak children with asthma and healthy children

In the study, 101 children with asthma of the Han nationality were enrolled, of which male accounted for 65.35%. The percentage of male was higher than female. There were 92 children in the control group, 48 boys and 44 girls. There were no significant differences between the two groups in the gender and age. 80 Kazakh children with asthma were enrolled in the group, including 48 males, and 32 females. And 93 children of Kazakh were included in the control group, 55 males and 38 females. There was no statistical difference between the two groups in terms of the gender and age in Kazakh (Table 2).

**Table 2:** The distribution of basic information in the Han and Kazak children with asthma and healthy children

Variable	Han nationality			Kazakh		
	Asthma	Control	P	Asthma	Control	*P*
Male (%)	66(65.35%)	48(52.17%)	0.063	48(60.00%)	55(59.14%)	0.908
Female (%)	35(34.65%)	44(47.83%)		32(40.00%)	38(40.86%)	
Age (y) (‾*x* ± *s*)	6.48 ± 2.38	5.74 ± 3.70	0.097	4.89 ± 3.63	4.98 ± 3.37	0.85

### The distribution of genotype frequency and allele frequency between asthma group and control group in Han children

A total of 193 Han children were included in the study, including 101 children in the asthma group and 92 children in the control group. There were significant differences in the distribution of the three genotype frequencies which including the gene loci ADRB2 rs1042713, IL4 rs2243250 and IL13 rs20541 between the asthma group and the control group. There was no statistical difference in the distribution of the genotype frequencies of FCER1B rs569108 between the asthma group and the control group. The proportion of ADRB2 rs1042713 A allele, IL4 rs2243250 T allele, FCER1B rs569108 A allele, and IL13 rs20541 G allele in the asthma group was higher than those in the control group. Except for FCER1B rs569108, the distribution of allele frequencies of other three genes in the asthma group and the control group were significantly different. See Table 3.

**Table 3:** The distribution of genotype frequency and allele frequency between asthma group and control group in Han children

Variable	Group	Genotype χ^2^ P					Allele χ^2^ P			
		AA	AG	GG			A	G		
*ADRB2* rs1042713	Asthma	28.71%	59.41%	11.88%	11.09	0.004	58.42%	41.58%	4.30	0.038
	Control	17.39%	52.17%	30.43%			43.48%	56.52%		
*IL4* rs2243250		TT	TC	CC			T	C		
	Asthma	70.30%	25.74%	3.96%	9.54	0.008	83.17%	16.83%	5.51	0.019
	conrtol	50.00%	38.04%	11.96%			69.02%	20.98%		
		AA	AG	GG			G	A		
*FCER1B*	Asthma	67.33%	27.72%	4.95%	2.16	0.34	16.83%	83.17%	1.19	0.276
rs569108	Control	76.09%	21.74%	2.17%			28.26%	71.74%		
		AA	AG	GG			G	A		
*IL13*	Asthma	11.88%	48.51%	39.61%	14.40	0.001	63.84%	36.16%	7.17	0.007
rs20541	Control	31.52%	47.83%	20.65%			44.47%	55.53%		

### The distribution of genotype frequency and allele frequency between the asthma group and the control group in Kazakh children

Among the Kazakh children, there were 80 children in the asthma group and 93 children in the control group. The genotype frequencies of ADRB2 rs1042713, IL4 rs2243250, FCER1B rs569108 and IL13 rs20541 were not significantly different between the asthma group and control group. The proportion of allele A in ADRB2 rs1042713, allele C in IL4 rs2243250, allele G in FCER1B rs569108, and allele G in IL13 rs20541 in the asthma group were higher than those in the control group, but there were no significant difference between the two groups. The results were shown in Table 4.

**Table 4:** The distribution of genotype frequency and allele frequency between asthma group and control group in Kazakh

Variable	Group	Genotype χ^2^ P					Allele χ^2^ P			
		AA	AG	GG			A	G		
*ADRB2 *rs1042713	Asthma	21.25%	43.75%	35.00%	0.79	0.67	43.13%	56.87%	0.52	0.47
	Control	17.20%	41.94%	40.86%			38.17%	61.83%		
*IL4 *rs2243250		TT	TC	CC			T	C		
	Asthma	22.50%	55.00%	22.50%	1.90	0.39	50.00%	50.00%	0.85	0.36
	Control	31.18%	51.61%	17.21%			56.99%	43.01%		
		AA	AG	GG			G	A		
*FCER1B*	Asthma	81.25%	16.25%	2.50%	1.38	0.49	10.62%	89.38%	0.70	0.40
rs569108	Control	87.10%	11.83%	1.07%			6.99%	93.01%		
		AA	AG	GG			G	A		
*IL13*	Asthma	11.25%	43.75%	45.00%	2.49	0.30	66.87%	33.13%	0.78	0.38
rs20541	Control	12.90%	53.76%	33.34%			60.22%	39.78%		

### The distribution of the different asthma risk genotypes in the Han and Kazak children

As were shown in Table 5, the high and low risk genotypes of asthma were classified according to the four different genotype combinations carried by each child. Among the Han children, the high-risk genotype of asthma accounted for 45.54% in the asthma group and 23.91% in the control group. The difference between the two groups was statistically significant. Among the Kazakh children, 16.67% of children in the asthma group were high-risk genotype, 83.33% were low-risk genotype. 17.20% of children in the control group were high-risk genotype, and the proportion of high-risk asthma children in the asthma group was lower than those in the control group.

**Table 5:** The distribution of children with different asthma risk genotypes between the asthma group and the normal group in the two nationality

Nationality	Group	High-risk (%)	Low-risk (%)	*P*	OR(95%*CI*)
Han	Asthma	45 (44.55%)	56 (55.45%)	0.003	2.56 (1.38–4.75)
	Control	22 (23.91%)	70 (76.09%)		
Kazak	Asthma	15 (16.67%)	65 (83.33%)	0.79	1.11 (0.51–2.42)
	Control	16 (17.20%)	77 (82.80%)		

OR means odds ratio, and CI means confidence interval

## Disscussion

There are significant differences in the race and region about the incidence of allergic diseases such as asthma in children, and the prevalence of asthma in different ethnic groups in the same region are also different. Similarly, the characteristics of many nationalities in China have led to the phenomenon that the prevalence of childhood asthma is different in different nationalities. In addition to environmental factor, the reasons for it are mostly believed to be related to genetic differences between different races or nationalities. At present, the research about the prevalence of allergic diseases among different races has been widely reported. A prospective birth cohort study in the UK found that children of Pakistani descent had more asthma diagnosed by doctors than Caucasians, and children of South Asian descent had the lowest prevalence of asthma [11]. In addition, the incidence of asthma and other atopic diseases in the African Americans are higher in developed countries, while the rate is lower in African countries. Some studies revealed that African lineages have more genetic variations of pro-inflammatory genes in order to adapt to the high infection burden in the environment in the evolutionary process [12, 13]. Because of the better health conditions in developed countries, these genetic variations of pro-inflammatory genes have become risk factors for asthma and atopic diseases [14]. A study from Brazil believed that more asthma in African Americans are non-atopic compared with native Americans, which may be due to the interaction between genetic and environmental factors [15].

Due to many nationalities in China, the genetic backgrounds of different races are also different, and the distribution of various ethnic groups has a certain degree of settlement, it is difficult to carry out genetic research on the difference of allergic diseases among different ethnic groups. The Kazak is a heterogeneous ethnic group with different blood and cultural backgrounds from the Han nationality. The Kazak people in China are mainly distributed in Ili and Altay regions of Xinjiang. Their lifestyles are mainly nomadic, and most of them are intermarriage, so their genetic lineage is relatively closed. In order to determine whether the four single nucleotide sites in our previous study are applicable to other ethnic groups, we selected Kazakh children in Xinjiang as the research object, and in order to eliminate the impact of environmental factors, we recruited the asthmatic children of Han nationality in Xinjiang who matched the sex and age of Kazakh children as the control factors. Through the case–control study, the distribution differences of the genotype frequencies and allele frequencies in the four SNP loci: ADRB2 rs1042713, IL4 rs2243250, FCER1B rs569108, IL13 rs20541in asthma group and control group. We found that: In the Han children, except for FCER1B rs569108, the SNP sites of the other three genes have significant differences between the asthma group and the control group, but in the Kazakh children, the distribution of different genotype frequencies and risk allele frequencies of the SNP sites of these four genes in the asthma group and the control group has no statistical difference. In addition, through different asthma risk classification for every child, we found that the proportion of high-risk asthma genotype in the asthma group of Han children was higher than those in the control group, and the high-risk asthma genotype was the risk factor in Han children, but the distribution of different asthma risk genotype in the Kazakh was not significantly different between the two groups. We consider that the possible reason is that the Kazakh children belong to the Tulan race, which is a mixed ethnic group of Mongolia and Caucasus, and is different from the genetic lineage of the Han nationality. Therefore, future research will explore the susceptibility gene polymorphism sites related to asthma in the Kazakh population.

The inherent phenotype and heterogeneity of childhood asthma are still challenges faced by many clinicians in the process of asthma diagnosis and management. Inherent phenotypes of asthma vary widely among different races, so what role does genetic variation play in it? At present, these are still unclear. Therefore, further research on the relationship between the SNP locus of asthma susceptibility gene and asthma phenotype among different races is needed in the future. In addition, only one minority nationality was included in this study, and more minorities should be included in the future to further clarify the application value of the four gene SNP in other nationalities.

## Conclusions

Therefore, we found that the four single nucleotide loci which including *ADRB2* rs1042713、*IL4* rs2243250、*FCER1B* rs569108、*IL13* rs20541 are suitable for Han children in Xinjiang, but not for the Kazakh children. It can be seen that the difference in the prevalence of asthma among children of different ethnic groups is closely related to genetic background.

## Data Availability

The datasets used and/or analysed during the current study are available from the corresponding author on reasonable request.

## Abbreviations

ADRB2: Beta2-adrenoreceptor
FCER1B: Beta subunit of the high affinity IgE receptor
SNP: Single nucleotide polymorphisms
PCR: Polymerase chain reaction
